# C_2_M: Configurable Chemical Middleware

**DOI:** 10.1002/cfg.124

**Published:** 2001-12

**Authors:** Paul van der Vet, Hans E. Roosendaal, Peter A. T. M. Geurts

**Affiliations:** 1 Department of Computer Science University of Twente Enschede The Netherlands; 2 Department of Computer Science and Department of Philosophy and Social Sciences University of Twente Enschede The Netherlands; 3 Department of Public Administration and Public Policy studies University of Twente Enschede The Netherlands; 4 Department of Computer Science University of Twente PO Box 217 Enschede 7500AE The Netherlands

## Abstract

One of the vexing problems that besets concurrent use of multiple, heterogeneous resources is format multiplicity. C_2_M aims to equip scientists with a wrapper generator on their desktop. The wrapper generator can build wrappers, or converters that can convert data from or into different formats, from a high-level description of the formats. The language in which such a high-level description is expressed is easy enough for scientists to be able to write format descriptions at minimal cost. In C_2_M, wrappers and documentation for human reading are automatically obtained from the same user-supplied specifications. Initial experiments demonstrate that the idea can, indeed, lead to the advent of usergoverned wrapper generators. Future research will consolidate the code and extend the approach to a realistic variety of formats.


**Comparative and Functional Genomics**
*Comp Funct Genom* 2001; **2**: 371–375.
**DOI:** 10.1002/cfg.124

**Review**

### $C_2M$: configurable chemical middleware

Paul van der Vet[1]*, Hans E. Roosendaal[2] and Peter A. T. M. Geurts[3]

[1] *Department of Computer Science, University of Twente, Enschede, the Netherlands*
[2] *Department of Computer Science and Department of Philosophy and Social Sciences, University of Twente, Enschede, the Netherlands*
[3] *Department of Public Administration and Public Policy Studies, University of Twente, Enschede, the Netherlands*

**Correspondence to:*
*Paul van der Vet, Department of Computer Science, University of Twente, PO Box 217, 7500AE Enschede, the Netherlands.*
*E-mail: vet@cs.utwente.nl*

Received: 29 October 2001
Accepted: 2 November 2001
Published online:
16 November 2001

### Abstract

**One of the vexing problems that besets concurrent use of multiple, heterogeneous resources is format multiplicity. $C_2M$ aims to equip scientists with a wrapper generator on their desktop. The wrapper generator can build wrappers, or converters that can convert data from or into different formats, from a high-level description of the formats. The language in which such a high-level description is expressed is easy enough for scientists to be able to write format descriptions at minimal cost. In $C_2M$, wrappers and documentation for human reading are automatically obtained from the same user-supplied specifications. Initial experiments demonstrate that the idea can, indeed, lead to the advent of user-governed wrapper generators. Future research will consolidate the code and extend the approach to a realistic variety of formats. Copyright © 2001 John Wiley & Sons, Ltd.**

**Keywords:** plaintext files; data integration; molecular structure files; format conversion

### Introduction

Plaintext databases constitute an important class of information stores. They can be used on every platform and most software has at least one way to read or write data in plaintext format. However, data processing and data integration are hindered because even within disciplines there is no uniform way to structure plaintext databases. Plaintext databases with molecular structure information, for example, exist in a wide variety of formats. Ironically, many of these formats have started their life as proposals for a standard format. Software for converting formats exists. For the domains considered here, chemical and biomolecular information, the Babel [2] system and SRS [11] are outstanding examples.

A more general approach to format conversion has been pioneered with the Chameleon system [9,10]. Chameleon, building on experiences in compiler design [1], is a system that reads specifications of the source and target formats to generate a converter or wrapper. Chameleon first converts the source file into data expressed in an intermediate format. In a second step, the data in the intermediate format are converted into the target format. This two-step approach is chosen because this way the specifications of the source and target formats can be written and stored separately. As a consequence, specifications are turned into separate parts of the converter program. Also, by a familiar argument [3], employing an intermediate format reduces the number of converters needed.

Today, the environment has changed compared to what it was when most formats and converters were designed. Information stored in databases is used for purposes format designers could not foresee. As more and more tasks are supported by software, data formats will multiply. Computing will increasingly be done in distributed environments, where many resources like databases and programs are available both in-house and remotely. Certain tasks may need data stemming from different sources to compare entries or to produce a file with merged information.

A type of task that will gain popularity in the near future involves interplay between a number of resources (whether in-house or remote), like data sources and programs to complete a particular task (Figure 1). Where, not so long ago, the ideal was

Copyright © 2001 John Wiley & Sons, Ltd.

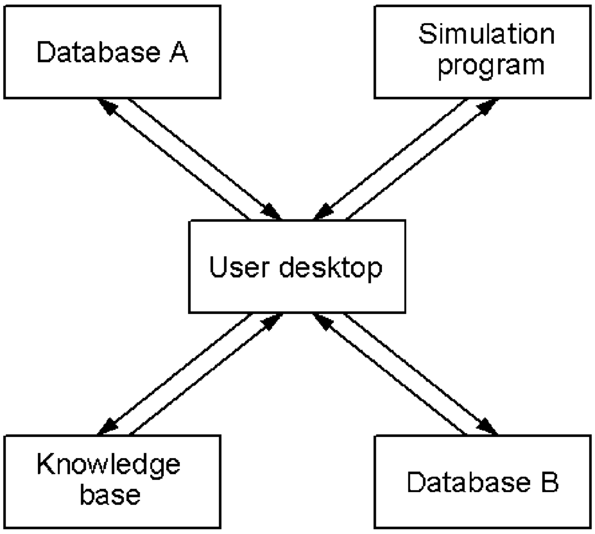

**Figure 1.** The environment: a federated information system or coalition. Resources can be in-house, or remote to be able to approach distributed data bases as a single federated database [5], we now face the challenge of federated information systems. Often, there is no further need for the configuration, once the task is done, and a different configuration will be set up to address a new task. The problem is that each resource will employ its own rules for input and output. At each interface between the user's desktop and a resource there is a conversion problem. Hard-coding a converter for every interface is time-consuming and error-prone. Worse, such pieces of code tend to become incomprehensible as time passes, so that edits prompted by small changes in the format are difficult, and eventually impossible.

Researchers will want to set up configurations with a limited lifetime at minimal cost, which rules out solutions like hard-coding. By the same token, standardisation becomes less adequate as a solution to the data interoperability problem because formats will multiply as tasks proliferate. Instead, middleware is needed that can be easily and quickly configured by a user to suit the needs at hand.

$C_2M$ builds on earlier efforts, in particular Chameleon, to present a design for data interoperability that attempts to cope with the demands of a constantly changing, distributed computing environment. One of the habitats for which it is typically designed is that of setting up middleware for configurations quickly and easily. Another typical use is that of quick construction of a filter to obtain only part of the information from a database, like only the annotations from a SWISS-PROT record [12]. Finally, it is foreseeable that some program could turn $C_2M$ specifications into conformance checkers that would be useful in preparing resources with fewer errors [7].

$C_2M$ is expressly designed to be middleware, rather than a general-purpose program. The data manipulations it can perform are limited to those that one can reasonably demand from converters that interface between a desktop application and external resources. The philosophy of $C_2M$ is to use it in a larger system such that each task is performed by the program best suited to it.

### The design of $C_2M$

The mode of operation of the current version of $C_2M$ is the following. Formats of external resources are specified in separate plaintext files that can be made and edited with any text editor. As for Chameleon, conversion is done in two steps using an intermediate format, called the *native format* in $C_2M$. The native format is also specified by the user in a separate plaintext file. Each format is known by a name that is unique within a $C_2M$ implementation. A program called the *code generator* turns each of these format specifications into a program module expressed in source code (Figure 2A). There is a pre-defined piece of source code that embodies the core of the converter. By compiling the modules and the core, one obtains a converter that can convert from and to each of the formats that have been specified (Figure 2B). Thus, a configuration as shown in Figure 1 needs a single $C_2M$ executable compiled from specifications of all formats used.

$C_2M$ enhances and extends the Chameleon approach in a number of ways.

(i) The native format is specified by the user in the form of a simple and purely declarative specification. There is no need to employ a single specification for a given domain (like molecular structure); setting up a native format is sufficiently easy to allow a native format for every particular task. The native format is a container for the data read from a foreign file. It is governed by a very simple data schema, or ontology [6]. Each $C_2M$ ontology defines a structure known as an annotated tree or (in artificial intelligence) a frame system.

Copyright © 2001 John Wiley & Sons, Ltd. 

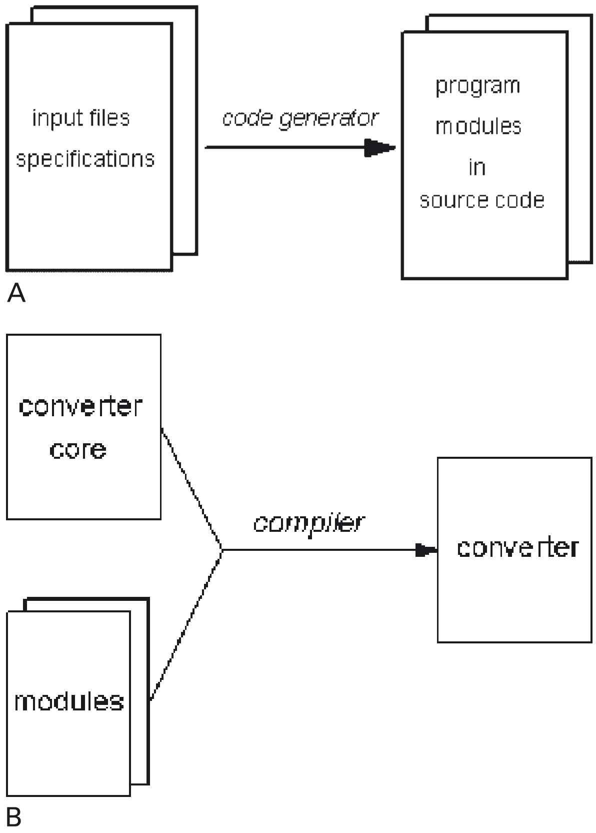

**Figure 2.** $C_2M$ mode of operation. To generate a converter: A) $C_2M$ generates source code program modules from every input file specification, B) and then compiles the modules with the core

There is minimal commitment so that interoperability, for example using the OKBC protocol [4], is minimally constrained.

(ii) $C_2M$'s native format can be set up to support operations on data other than simple conversion, like producing a target file that contains merged data from different, heterogeneous files or comparing such data, for instance to identify potential errors.

(iii) A special declarative language is used for file format specifications that abstracts from a particular implementation. The language can even be used as a means to communicate a file format without having the intention to generate a converter. The conventions for structuring a format specification are dictated by the particular demands of the code generator and by agreements between parties that want to exchange specifications. If specifications are used purely locally, only the local implementation of the code generator remains as a source of conventions. When exchange is desired, the degree of standardisation of conventions is still minimal.

(iv) A specification of a file format consists of two parts. The first, syntactic part specifies the structure of the file. A plaintext data file is a sequence of strings. (The notion of string here covers both multi-character strings and strings that consist of a single character, say, a space character.) Strings fall into one of two categories. The first category, that of *meaningful strings*, comprises the strings that hold the fields, in other words, the information proper. The second category, that of *landmarks*, embraces all strings that serve to identify and delimit records and fields. The function of identifier and delimiter is often combined. Landmarks may identify and/or delimit groupings like the sequence of fields that hold atom information in a record that describes a molecular structure. The second, semantic part of a file format specification maps the information as it is expressed in the file onto the form determined by the native format. This makes division of work possible, for instance the content provider offers the syntactic part while the user adds the semantic part. By means of an appropriate combination of a native format and the semantic part of a file specification, significant efficiency gains can be obtained.

(v) Analysis of source files proceeds down to the level of individual characters, so that there are minimal restrictions on the kind of formats. Technically, analysis proceeds down to the level of individual bytes, although the conventions we have employed in our experiments have not yet made this explicit. The consequence is that a simple extension of the conventions allows $C_2M$ to handle control characters or Unicode without sacrificing the simplicity of the language. In other words, files other than plaintext files can be read and written using the same design.

(vi) Extra constructs have been added to allow the user to define his own character classes and to handle constructions stemming from the Fortran era, such as having to process a fixed number of lines where the actual number has to be read from the source file itself.

(vii) Specifications of formats (whether a file or

Copyright © 2001 John Wiley & Sons, Ltd. 

native format) incorporate ample documentation to facilitate maintenance. Specifications adhere to Knuth's principle of literate programming [8], meaning that code for the program proper and human-readable documentation are produced from the same source specification. This way, the documentation always applies to the code as it is actually executed.

### Experiments

We are conducting experiments to assess the viability of the approach. So far, the experiments are confined to the converter functionality. Conversion is done in three parts. Reading involves a parsing step governed by the syntactic part of a file format specification, followed by a semantic step governed by the semantic part of a file format specification (Figure 3). Writing is done in a single step, where syntactic and semantic parts are used together (Figure 4). We have concentrated on converters for plaintext files with molecular structure information. The source files with molecular structure information were prepared by drawing structures with the help of the ChemDraw package. Using ChemDraw's export facility, these drawings were exported as plaintext files in a number of formats. The files were then converted by $C_2M$, and the resulting plaintext files were imported into ChemDraw to ascertain that the conversion was correct. So far, we have only experimented with a few formats. The results are promising but we also have identified issues that have to be addressed in the course of further development.

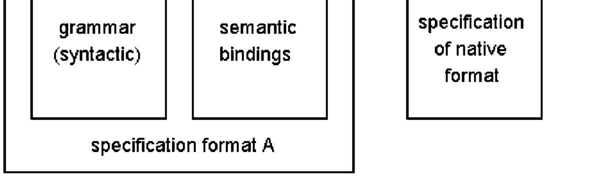

**Figure 3.** $C_2M$ conversion: reading. Reading is done in two steps, corresponding to grammar and semantic bindings

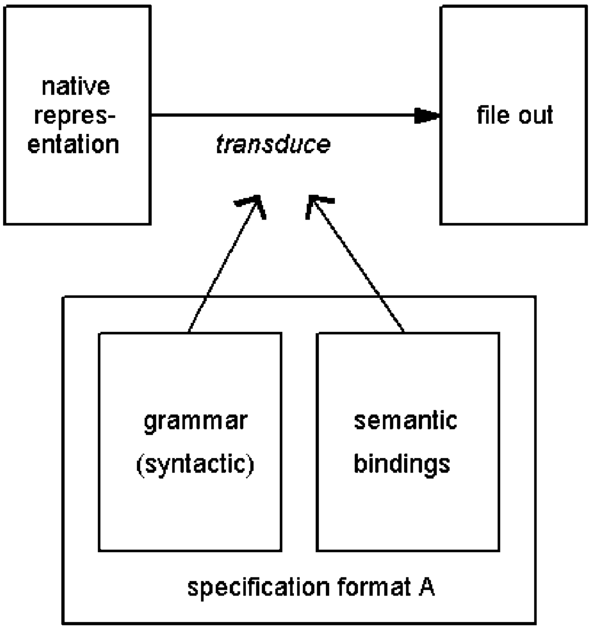

**Figure 4.** $C_2M$ conversion: writing. Writing is done in a single step. The specifications for reading are re-used to whatever extent is possible

Our current implementation uses Prolog as implementation language. We used the Quintus Prolog implementation because it is one of the fastest on the market. As a disadvantage, Quintus code is portable over Windows and Unix platforms, but not, for instance, over Mac platforms. We found that conversion of a simple source file with structure information for a molecule with a total of 1000 atoms and bonds into an equally simple target file takes between 1.2 and 1.6 seconds, depending on the complexity of the file. We have tentatively concluded that scaling behaviour as the source file grows appears to be linear. This is sufficiently fast for many applications. As we expected, the parsing step is the rate-determining step of the whole process. Significant efficiency gains are possible even when Prolog remains the programming platform.

### Discussion

Preliminary experiments indicate that the $C_2M$ approach presents a viable way to address problems of conversion. We now want to explore a greater

Copyright © 2001 John Wiley & Sons, Ltd. 

variety of formats to come up with a version that can be put to real-life tasks. In the course of our experiments, we have noted a number of points for future research.

The main problem we are working on now involves the so-called *instantiation functions*. These functions map strings as they are found in a file onto representations that conform to native format while reading, or *vice versa* while writing. Instantiation functions have to be hard-coded into the predefined core of the converter. This means that a user cannot extend the set of instantiation functions in a way other than combining existing functions. It is not clear what criteria must be used to assess the value of an existing set of instantiation functions because $C_2M$ is designed for a distributed environment and there may well be other programs that can handle difficult mapping tasks. For example, an elaborate set of functions for numbers and dates is an obvious candidate for inclusion in $C_2M$ (but co-ordinate transformation is another case). There are mathematical packages on the market that perform co-ordinate transformations efficiently by means of built-in routines. Duplication of these routines within $C_2M$ is of doubtful value. In the longer term, only familiarity with a great variety of formats will indicate which set is satisfactory.

Another point of some concern is efficiency. The current implementation of the code generator is not optimised for speed. Code generation takes several seconds for each user specification. The process can be performed significantly faster. The generator does, however, incorporate measures to enhance the efficiency of the converter.

Finally, we want to extend $C_2M$ to handle merging and comparison tasks in addition to conversion tasks. As we explained above, $C_2M$ is in principle equipped to handle these tasks because they operate on a common native format. The first step would be to define a language that enables a user to specify merging and comparison tasks. The second step would then be to extend the code generator and the core program to implement these tasks.

In sum, we believe that $C_2M$ paves the way for a flexible system able to convert, merge and compare data in a web-based, rapidly changing environment.

### References

1. Aho AV, Ullman JD. 1979. *Principles of Compiler Design*. Addison-Wesley: Reading MA
2. Babel: **http://smog.com/chem/babel/**
3. Barnard JM. 1990. *J Chem Inf Comp Sci* **30**: 81–96.
4. Chaudri VK, Farquhar A, Fikes R, Karp PD, Rice JP. 1998. OKBC: A programmatic foundation for knowledge base interoperability. In *Proceedings of the 15th National Conference on Artificial Intelligence (AAAI-98)*, Mostow J, Rich C (eds). AAAI Press: Menlo Park, CA; 600–607.
5. Gray PMD, Kemp GJL. 2000. Federated database technology for data integration: lessons from bioinformatics. In *Electronic Collaboration in Science*, Koslow SH, Huerta MF (eds). Lawrence Erlbaum: Mahwah NJ; 45–72.
6. Gruber TR. 1993. A translation approach to portable ontology specifications. *Knowledge Acquisition* **5**: 199–220.
7. Karp PD. 2001. Many Genbank entries for complete microbial genomes violate the Genbank standard. *Comp Funct Genom* **2**: 25–27.
8. Knuth DE. 1992. *Literate Programming*. Center for the Study of Language and Information, Stanford University: Palo Alto, CA
9. Mamrak SA, Kaelbling MJ, Nicholas CK, Share M. 1989. Chameleon: a system for solving the data-translation problem. *IEEE Transactions on Software Engineering* **15**: 1090–1108.
10. Mamrak SA, O'Connell CS, Barnes J. 1994. *The Integrated Chameleon Architectures*. Prentice Hall: Englewood Cliffs, NJ.
11. SRS: **http://www.cmbi.kun.nl/srs6** or **http://srs.ebi.ac.uk**
12. SWISS-PROT: **http://www.expasy.ch/sprot/sprot-top.html**

Copyright © 2001 John Wiley & Sons, Ltd. 
